# Higher dietary phytochemical index is associated with lower odds of infertility: a case–control study

**DOI:** 10.3389/fnut.2026.1865952

**Published:** 2026-07-10

**Authors:** Yanfei Ji, Hui Zhao

**Affiliations:** Department of Obstetrics and Gynecology, The Second Hospital of Shanxi Medical University, Taiyuan, Shanxi Province, China

**Keywords:** anti-Müllerian hormone, case–control study, dietary phytochemical index, infertility, inflammation, insulin resistance

## Abstract

**Background:**

Dietary phytochemicals possess antioxidant and anti-inflammatory properties, yet their relationship with female infertility remains unclear. We examined the association between the Dietary Phytochemical Index (DPI) and infertility, and whether oxidative stress/inflammatory biomarkers mediate this link.

**Methods:**

This case–control study included 400 women (200 infertile, 200 fertile). Cases were clinically diagnosed infertility patients; controls had ≥1 live birth. DPI was calculated from food-frequency questionnaires. Fasting biomarkers (MDA, HOMA-IR, hs-CRP, IL-6, TNF-*α*, TAC) were measured. Logistic regression evaluated DPI–infertility associations, and bootstrap mediation analysis tested indirect effects through biomarkers.

**Results:**

Infertile women had significantly lower DPI (28.2 ± 8.4 vs. 34.1 ± 9.8, *p* < 0.001). Each 1 unit increase in DPI was associated with 7% lower odds of infertility (OR 0.931, 95% CI: 0.909–0.953). Expressed per 10 unit increment (a more clinically interpretable change), this corresponds to approximately 48% lower odds (OR 0.48, 95% CI: 0.39–0.59). Women in the highest DPI tertile had 80% lower odds than those in the lowest (OR 0.205, 95% CI 0.119–0.352), with a significant dose–response trend (*p* < 0.001). DPI was inversely associated with MDA, HOMA-IR, hs-CRP, and IL-6 (all *p* < 0.05), but none of the examined biomarkers significantly mediated the DPI–infertility association (all indirect effect 95% CIs included zero; maximum proportion mediated: 12.6% for HOMA-IR). The direct effect of DPI remained significant in all models.

**Conclusion:**

Higher dietary phytochemical intake shows a strong independent association with lower infertility odds in this exploratory case–control study. Causal inference is precluded by design limitations. In exploratory mediation analyses, circulating oxidative stress and inflammatory biomarkers did not significantly mediate this association; however, this finding does not exclude local tissue level or alternative mechanistic pathways. Prospective studies are needed to establish causality and elucidate mechanisms.

## Introduction

Infertility, defined as the failure to achieve clinical pregnancy after 12 months of regular unprotected intercourse, affects an estimated 10–15% of couples globally and constitutes a significant public health concern (1–3). Beyond the psychological and social distress experienced by affected individuals, infertility imposes substantial economic burdens on healthcare systems and families (3). While advanced reproductive technologies have expanded therapeutic options, success rates are constrained by both non-modifiable factors—most notably maternal age and ovarian reserve—and modifiable lifestyle determinants (4). Consequently, the identification of modifiable risk factors amenable to preconception intervention has emerged as a research priority. Metabolic disturbances, particularly insulin resistance, further exacerbate reproductive dysfunction by disrupting steroidogenesis and ovarian signaling pathways (5). These mechanisms are especially relevant even in women without overt polycystic ovary syndrome, indicating a broader metabolic contribution to infertility (6, 7).

Among modifiable exposures, dietary patterns have garnered escalating scientific interest (8, 9). Epidemiological evidence increasingly supports the notion that plant-rich dietary patterns confer a protective advantage against infertility, whereas Western-style diets characterized by high intakes of processed foods, refined carbohydrates, and saturated fats appear to elevate risk (10, 11). Adherence to a Mediterranean diet, for instance, has been associated with improved pregnancy rates in women undergoing assisted reproductive technology (12). Nevertheless, the specific bioactive constituents responsible for these observed associations remain incompletely characterized, prompting growing interest in the role of phytochemicals—naturally occurring, plant-derived compounds with potent antioxidant, anti-inflammatory, and insulin-sensitizing properties.

Phytochemicals, a chemically diverse group encompassing carotenoids, flavonoids, polyphenols, and organosulfur compounds, are derived almost exclusively from plant-based foods such as fruits, vegetables, whole grains, legumes, nuts, and seeds (10, 13). The DPI, introduced by McCarty as a quantitative metric to capture the overall phytochemical density of the diet, is calculated as the percentage of total daily energy intake derived from phytochemical-rich food sources (14). This composite index offers an integrative approach to assessing dietary phytochemical exposure, circumventing the limitations inherent in focusing on isolated nutrients. We selected DPI over alternative indices (Mediterranean Diet Score, HEI, DII) for three reasons. First, DPI directly measures phytochemical density, whereas the Mediterranean Diet Score and HEI capture broader dietary patterns. Second, the DII reflects inflammatory potential but not phytochemical-specific pathways (e.g., mitochondrial function, hormone metabolism) (15). Third, DPI is a simple, energy-adjusted metric calculable from standard FFQ data, enhancing reproducibility (16). A growing literature supports DPI’s relevance in reproductive health (17). A growing body of literature supports the salience of the DPI in reproductive contexts. In female reproductive health, a cross sectional study among women undergoing *in vitro* fertilization reported that higher DPI was significantly associated with increased rates of ongoing pregnancy, suggesting benefits at the level of embryo implantation and early development (18). In male reproductive health, a hospital based case–control study demonstrated that greater dietary consumption of specific carotenoids was inversely associated with the risk of teratozoospermia, indicating that phytochemicals may also improve sperm morphology and function (19). Extending these observations to female infertility specifically, the present study investigates whether higher DPI is associated with lower odds of clinically diagnosed infertility—a question that has not been directly examined in prior research. Collectively, these observations suggest that a phytochemical-rich diet may represent a modifiable protective factor across multiple aspects of reproductive health. Phytochemical rich diets exert biological effects through multiple interconnected pathways relevant to female fertility. First, phytochemicals directly scavenge reactive oxygen species (ROS) and upregulate endogenous antioxidant defenses, thereby protecting oocytes from oxidative damage that impairs meiotic competence and embryo developmental potential. Second, these compounds modulate mitochondrial function by preserving mitochondrial membrane potential, reducing cytochrome c release, and enhancing ATP production, which is critical for oocyte maturation and fertilization. Third, phytochemicals influence folliculogenesis and oocyte maturation through signaling pathways including AMPK, PI3K/Akt, and Nrf2, which regulate granulosa cell proliferation, steroidogenesis, and apoptosis. Fourth, emerging evidence indicates that gut microbiota metabolize dietary polyphenols into bioactive compounds (e.g., urolithins, equol) that may influence endometrial receptivity and implantation through estrogen receptor modulation and antiinflammatory actions in the reproductive tract. Collectively, these pathways provide a mechanistic framework linking phytochemical rich diets to improved female fertility outcomes (4, 10, 14, 18). Despite these mechanistic links, epidemiological evidence examining comprehensive phytochemical exposure using indices such as the DPI remains limited and inconsistent.

Despite these encouraging findings, critical knowledge gaps remain. Most studies have examined dietary indices in relation to ART outcomes rather than infertility itself, and no study has simultaneously examined inflammation, oxidative stress, and insulin resistance as mediators. Furthermore, observational nutritional epidemiology has inherent limitations: dietary assessment is subject to measurement error and recall bias; residual confounding from unmeasured lifestyle factors (e.g., health consciousness, physical activity) may persist despite adjustment; and distinguishing specific dietary components from correlated healthy behaviors is challenging. These limitations constrain causal inference and underscore the need for prospective studies with repeated dietary measurements (20, 21). Furthermore, the relationship between the DPI and ovarian reserve markers such as anti-Müllerian hormone (AMH) and antral follicle count (AFC) remains largely unexplored, with conflicting evidence regarding dietary phytoestrogen intake.

To address these knowledge gaps, the present study was designed with three specific objectives and corresponding *a priori* hypotheses. First, we evaluated the independent association between DPI and infertility risk, hypothesizing that higher DPI would be associated with lower odds of infertility in a dose–response manner. Second, we tested whether oxidative stress, inflammatory, and insulin resistance biomarkers mediate this association, hypothesizing that the protective effect of DPI would be partially explained by lower circulating levels of MDA, HOMAIR, hsCRP, and IL6 among women with higher DPI. Third, we explored cross sectional associations between DPI and ovarian reserve markers (AMH, AFC) as exploratory outcomes, without a directional hypothesis given the limited prior evidence.

## Methods

### Study design and setting

This case–control study was conducted between January 2025 and March 2026 at the Department of Obstetrics and Gynecology of the Second Hospital of Shanxi Medical University, a tertiary care academic medical center in Taiyuan, Shanxi Province, China. The study protocol was designed and is reported in accordance with the Strengthening the Reporting of Observational Studies in Epidemiology (STROBE) guidelines for case–control studies. Ethics approval for this study (approval no. 2025yx463) was provided by the ethics committee of the Second Hospital of Shanxi Medical University, China. All procedures performed were in accordance with the ethical standards of the institutional research committee and with the 1964 Helsinki declaration and its later amendments. Written informed consent was obtained from all individual participants included in the study.

### Participants and case definition

A total of 400 women aged 20–45 years were enrolled, consisting of 200 infertile women designated as cases and 200 fertile women designated as controls. Cases were defined as women who had failed to achieve a clinical pregnancy after 12 months or more of regular unprotected sexual intercourse, in accordance with the criteria established by the International Committee for Monitoring Assisted Reproductive Technology and the WHO (22). Cases were recruited consecutively from the outpatient infertility clinic of the participating hospital. Infertility etiology was categorized using standardized criteria. PCOS was diagnosed by Rotterdam criteria (≥2 of: oligo-anovulation [cycles >35 days or progesterone <3 ng/mL], clinical/biochemical hyperandrogenism, or polycystic ovarian morphology on ultrasound). Ovulatory dysfunction (non-PCOS) was defined by luteal progesterone <3 ng/mL or menstrual abnormalities without hyperandrogenism or polycystic ovaries. Unexplained infertility required normal semen analysis, ovulation, tubal patency, and uterine cavity. Other causes included endometriosis stage I–II or cervical factor. Controls were recruited from the same geographic area via community advertisements and routine gynecologic visits. Controls were frequency-matched to cases by age (±2 years) and required proven fertility (≥1 live birth). This criterion ensures cases and controls are clearly distinguished but may exclude nulliparous fertile women and may include women with undiagnosed subfertility who nevertheless achieved pregnancy.

**Figure 1:**
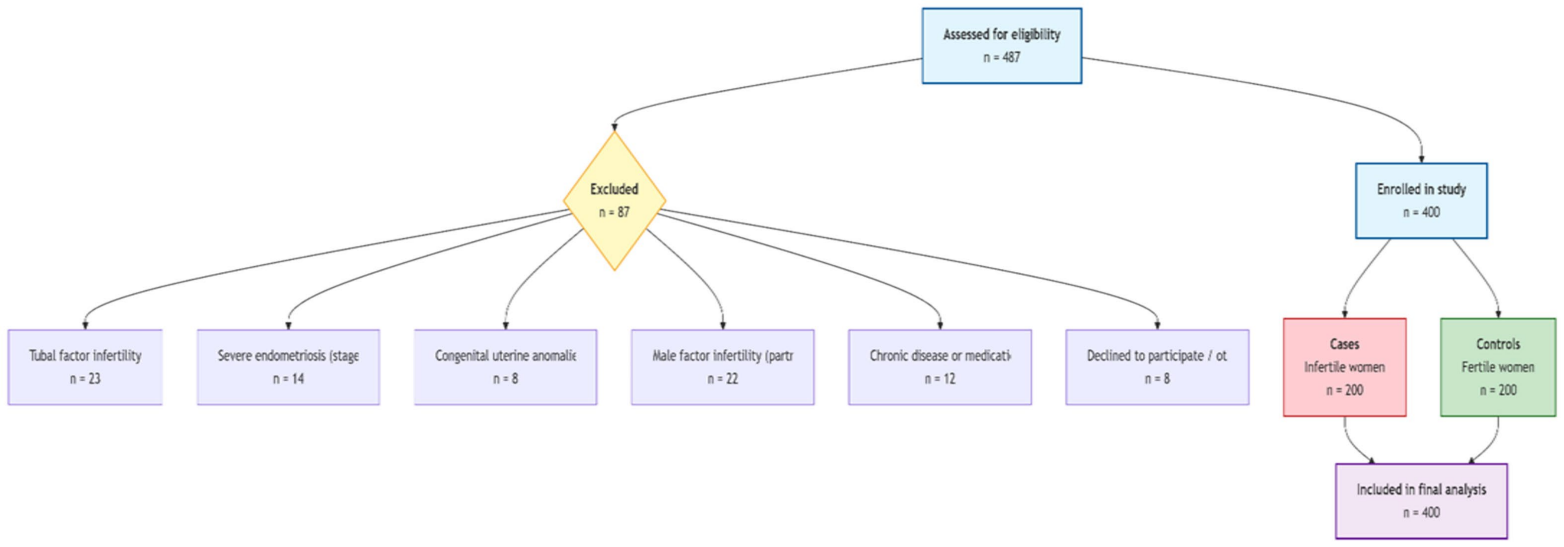
Flow diagram of participant recruitment, eligibility assessment, and enrollment in the case–control study.

Women were excluded from participation if they met any of the following criteria: documented tubal factor infertility as confirmed by hysterosalpingography or diagnostic laparoscopy; severe endometriosis classified as stage III or IV according to the revised criteria of the American Society for Reproductive Medicine; congenital uterine anomalies diagnosed by imaging; male factor infertility defined by a partner with semen parameters falling below the fifth percentile of World Health Organization reference values; a history of malignancy; current or recent pregnancy or lactation within the preceding 6 months; use of hormonal contraception or intrauterine devices within the preceding 3 months; use of medications known to affect reproductive hormones or metabolic parameters including insulin sensitizers, lipid-lowering agents, or corticosteroids; and self-reported chronic diseases including diabetes mellitus, thyroid disorders, cardiovascular disease, renal impairment, or hepatic dysfunction. We excluded tubal factor and severe endometriosis (stage III–IV) to reduce etiological heterogeneity. Tubal infertility is primarily mechanical and unlikely diet-responsive, while severe endometriosis involves extensive anatomical distortion that may overwhelm dietary effects. This limits generalizability to these subtypes; our findings apply mainly to infertility mediated by ovulatory, endocrine, or unexplained mechanisms. Of the initial pool, 87 women were excluded: 23 with tubal factor infertility, 14 with severe endometriosis, 8 with congenital uterine anomalies, 22 with male factor infertility, 12 with chronic diseases or medication use, and 8 who declined participation. The remaining 400 women (200 infertile cases, 200 fertile controls) were enrolled. Refusers (*n* = 8) cited lack of time and disinterest. Figure 1 shows the enrollment flowchart.

### Sample size considerations

The sample size for this study was determined *a priori* based on the ability to detect a clinically meaningful difference in the primary exposure variable, the Dietary Phytochemical Index, between infertile and fertile women. Based on previously published data examining dietary indices in reproductive health research, the standard deviation of the DPI in the target population was assumed to be approximately 9 units. With 200 cases and 200 controls, the study had 80% power (*α* = 0.05, two-sided) to detect: (1) a mean DPI difference of 2.5 units between groups (SD ≈ 9 units; Cohen’s *d* = 0.28), and (2) an odds ratio of ≥1.8 for infertility comparing extreme DPI tertiles (i.e., a 1.8-fold increased risk). The observed protective association (OR ≈0.2) is in the opposite direction and of larger magnitude than anticipated; *post hoc* power for detecting an OR of 0.2 is >99%, but the *a priori* calculation assumed a risk increase, not a protective effect, assuming 30% exposure prevalence in controls. Additionally, the sample size provided ≥10 outcome events per variable included in multivariable logistic regression models, ensuring model stability (23).

### Data collection and variables

Data collection was performed by trained research staff using standardized operating procedures and validated instruments. All interviewers completed a structured training program covering questionnaire administration, anthropometric measurement techniques, and ethical considerations in human subject’s research. To minimize inter-observer variability, anthropometric measurements were performed by the same two trained technicians throughout the study period.

Demographic and lifestyle characteristics were collected via structured face-to-face interviews. Age was recorded in completed years at the time of enrollment. Socioeconomic status was self-reported on an ordinal scale ranging from one to five, with higher scores indicating greater socioeconomic advantage. Educational attainment was documented as total years of formal education completed. Income level was categorized into four ordinal levels based on self-reported monthly household income. Smoking status was dichotomized as current smoker versus non-smoker. Dietary supplement use was recorded as any regular consumption of vitamin, mineral, or herbal supplements versus none.

Anthropometric measurements were obtained with participants wearing lightweight indoor clothing without shoes. Body weight was measured to the nearest 0.1 kilogram using a calibrated digital scale. Standing height was measured to the nearest 0.1 centimeter using a wall-mounted stadiometer with the participant positioned in the Frankfort horizontal plane. Body mass index was calculated as weight in kilograms divided by the square of height in meters. Waist circumference was measured at the approximate midpoint between the lowest palpable rib and the superior border of the iliac crest using a non-stretchable flexible measuring tape, with the measurement taken at the end of a normal expiration and recorded to the nearest 0.1 centimeter. Physical activity was assessed using the short form of the International Physical Activity Questionnaire and was expressed as metabolic equivalent of task minutes per week.

### Dietary assessment and dietary phytochemical index

Habitual dietary intake over the 12 months preceding enrollment was assessed using a validated 147-item semi-quantitative food frequency questionnaire that was adapted specifically for use in Chinese populations. The original questionnaire was previously validated against multiple 24-h dietary recalls (three nonconsecutive days, including one weekend day) in a comparable Chinese cohort of women of reproductive age (range 20–45 years), demonstrating correlation coefficients ranging from 0.52 to 0.68 for major food groups and attenuation corrected correlation coefficients of 0.60–0.75. The validation study specifically included women with both proven fertility and infertility to ensure comparability. Nevertheless, recall bias is an inherent limitation of FFQ based dietary assessment; participants may differentially recall or report dietary intake based on their fertility status, and retrospective assessment over 12 months is subject to imperfect recall. We addressed this by using standardized food models and portion size photographs during face to face interviews to enhance accuracy, and we acknowledge residual recall bias as a potential limitation. The questionnaire captured the usual frequency of consumption of each food item, with response options ranging from never or less than once per month to six or more times per day, along with the typical portion size consumed. Trained interviewers administered the questionnaire during face-to-face sessions, using standardized food models and portion size photographs to enhance the accuracy of portion size estimation. Daily intakes of total energy, macronutrients, dietary fiber, and micronutrients including vitamins C and E, beta-carotene, and lutein were computed by linking the food frequency questionnaire data to the Chinese Food Composition Database.

The DPI was calculated in accordance with the methodology originally proposed by McCarty. The total energy intake derived from phytochemical-rich food sources—operationally defined as fruits, vegetables, legumes, whole grains, nuts, seeds, and olive oil—was summed and then divided by the total daily energy intake derived from all dietary sources. Given cultural dietary patterns, olive oil intake was minimal and did not substantially influence DPI calculation. However, the DPI was originally developed in Western populations; its performance in Chinese populations, where phytochemical sources differ (e.g., soy, tea, diverse vegetables), has not been formally validated. This may affect the index’s ability to capture total phytochemical exposure in our sample. This quotient was multiplied by 100 to express the DPI as the percentage of total daily energy contributed by phytochemical-rich foods. Higher DPI values therefore indicate a greater proportion of dietary energy derived from phytochemically dense plant foods (14). However, DPI does not capture phytochemical-dense foods with negligible caloric content (e.g., tea, coffee, herbs, spices), which may lead to underestimation of total phytochemical exposure—a limitation to consider when interpreting the observed associations.

### Blood collection and laboratory analyses

Fasting venous blood samples of approximately 10 milliliters were collected from all participants during the early follicular phase of the menstrual cycle, specifically between cycle days two and five, following a confirmed overnight fast of at least 10 h. For anovulatory or oligo-ovulatory women, blood samples were collected on a random day following a confirmed progesterone level <1 ng/mL to ensure a follicular-phase hormonal environment, or after a progesterone-induced withdrawal bleed. Blood samples were drawn into serum separator tubes and tubes containing ethylenediaminetetraacetic acid as an anticoagulant. Samples were allowed to clot at ambient room temperature for 30 min when appropriate and were subsequently centrifuged at 3000 rpm (≈500 ×*g*) for 10 min at 4 degrees Celsius. Aliquots of serum and plasma were prepared and immediately stored at −80 °C pending batch analysis to ensure analytic stability.

### Biochemical and hormonal assays

Fasting serum glucose was measured using the glucose oxidase method on an automated clinical chemistry analyzer (units: mg/dL). Fasting serum insulin was quantified by chemiluminescent immunoassay (units: μIU/mL). Insulin resistance was estimated using HOMA-IR, calculated as [glucose (mg/dL) × insulin (μIU/mL)]/405. Inflammatory biomarkers including high-sensitivity C-reactive protein (hs-CRP, units: mg/L) and tumor necrosis factor-alpha (TNF-*α*, units: pg./mL) were measured using commercially available ELISA kits. Serum insulin, interleukin-6 (IL-6, units: pg./mL), and anti-Müllerian hormone (AMH, units: ng/mL) were quantified using ELISA kits from Monobind Inc. (Lake Forest, CA, USA). hs-CRP was measured using a high-sensitivity ELISA kit from Diagnostics Biochem Canada Inc. (DBC, Ontario, Canada); detection limit: 0.1 mg/L. Total antioxidant capacity (TAC, units: mmol/L) and malondialdehyde (MDA, units: μmol/L) were determined using kits from ZellBio GmbH (Ulm, Germany); detection limits: TAC 0.05 mmol/L, MDA 0.1 μmol/L. FSH (IU/L), LH (IU/L), estradiol (pmol/L), progesterone (nmol/L), and sex hormone-binding globulin (SHBG, nmol/L) were measured using ELISA kits from DRG Instruments GmbH (Marburg, Germany); detection limits: FSH 0.1 IU/L, LH 0.1 IU/L, estradiol 10 pmol/L, progesterone 0.1 nmol/L, SHBG 0.5 nmol/L. Calibration was performed using manufacturer-provided calibrators and three-level quality control samples (low, medium, high) in each plate. All assays were performed according to manufacturer protocols. Laboratory personnel were blinded to participants’ group status. All biochemical assays were performed in duplicate, and intra-assay and inter-assay coefficients of variation were maintained below 10% for all analyses. The free androgen index was calculated as [total testosterone (nmol/L)/SHBG (nmol/L)] × 100.

### Ultrasound assessment of ovarian reserve

Transvaginal ultrasonography was performed by a single experienced gynecologist who was blinded to the participants’ case or control status. All examinations were conducted during the early follicular phase of the menstrual cycle, specifically between cycle days two and five, to ensure standardized assessment of ovarian reserve. A high-frequency endovaginally transducer was used to visualize both ovaries in the sagittal and coronal planes. The total number of antral follicles measuring between 2 and 10 millimeters in mean diameter in both ovaries was carefully counted and recorded as the antral follicle count, which served as the primary ultrasound-derived indicator of ovarian reserve for the study.

### Statistical analysis

**Figure 2:**
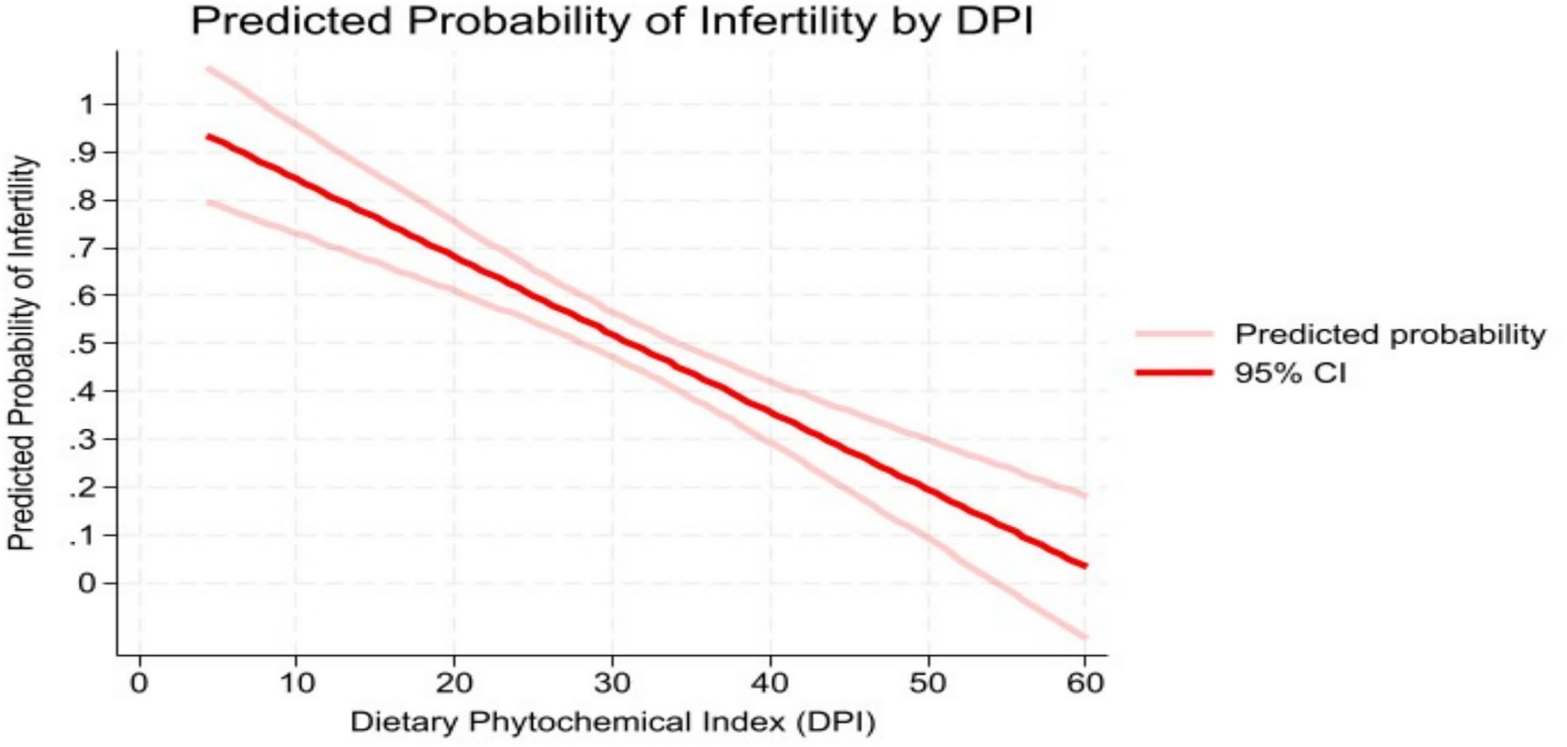
Adjusted dose–response relationship between the Dietary Phytochemical Index (DPI) and the predicted probability of infertility.

Continuous variables were assessed for normality using the Shapiro–Wilk test; normally distributed data are presented as mean ± SD and compared by independent *t*-tests, skewed variables as median (IQR) with Mann–Whitney U tests. Skewed biomarkers were natural log-transformed prior to regression analyses. Categorical variables are n (%) compared by *χ*^2^ or Fisher’s exact test. Participants were categorized into DPI tertiles based on the overall sample distribution. Linear trends across DPI tertiles were tested by assigning tertile-specific median DPI values (calculated separately for fertile and infertile groups) as continuous predictors in regression models stratified by fertility status. This avoids circularity inherent in using overall sample medians for subgroup analyses. Multivariable logistic regression estimated ORs and 95% CIs for infertility across DPI tertiles (lowest tertile as reference). Model 1 was unadjusted; Model 2 adjusted for age, BMI, total energy intake, physical activity, smoking, and supplement use. The linearity of continuous predictors (DPI, age, BMI, energy, physical activity) was verified by comparing linear models to those with restricted cubic splines (3 knots); likelihood ratio tests showed no deviation from linearity (all *p* > 0.10). Model calibration was assessed by the Hosmer–Lemeshow test, and discrimination by the AUC with bootstrap validation (200 resamples). Fully adjusted linear regression models (using Model 2 covariates) were fit separately for fertile and infertile women to examine cross-sectional associations between continuous DPI and each biomarker. Ovarian reserve markers (AMH, AFC) were exploratory outcomes (Table 1 baseline comparisons only). Mediation analysis quantified indirect effects of DPI on infertility through HOMA-IR, hs-CRP, and MDA. Indirect effects = (path a from linear regression) × (path b from logistic regression) under full Model 2 adjustment, with 95% CIs derived by the delta method (1,000 bootstrap replications). Mediation assumptions (temporal ordering, no unmeasured confounding, no measurement error) were considered; due to the cross-sectional design, results are exploratory, not causal. VIFs were examined (all <2, no multicollinearity). Missing data <5% for all variables; complete-case analysis was primary. Sensitivity analyses excluding smokers and BMI ≥ 30 kg/m^2^ yielded similar results. Multiple imputation by chained equations (20 imputed datasets) gave materially unchanged results (available upon request). Given the exploratory nature of biomarker analyses (Table 2), we did not adjust for multiple comparisons to avoid inflating Type II error; however, readers should interpret these findings cautiously, as the nominal *p*-values do not account for the number of comparisons performed. The finding for IL-6 (*p* = 0.030) may represent a false positive, and confirmation in independent cohorts is needed. Adjusted predicted probabilities of infertility (Figure 2) were estimated from the fully adjusted logistic regression model (Model 3), with continuous covariates held at their means and categorical covariates at reference categories (non-smoking, no supplement use, SES level 3). Linearity of DPI was verified using restricted cubic splines (three knots); the likelihood ratio test comparing linear vs. spline models was non-significant (*p* = 0.23). All analyses used Stata 17.0 with two-tailed *p* < 0.05 considered significant.

**Table 1:** Baseline characteristics by infertility status.

Characteristic	Total (*n* = 400)	Fertile (*n* = 200)	Infertile (*n* = 200)	*p*-value
Continuous variables				
Age (years)	31.7 ± 5.0	31.4 ± 4.6	32.0 ± 5.3	0.235
BMI (kg/m^2^)	27.0 ± 4.0	26.4 ± 3.8	27.5 ± 4.1	**0.006**
Waist circumference (cm)	89.0 ± 9.8	87.3 ± 9.2	90.6 ± 10.1	**<0.001**
Physical activity (MET-min/week)	1502.7 ± 488.7	1485.1 ± 493.7	1520.4 ± 484.3	0.471
DPI	31.2 ± 9.6	34.1 ± 9.8	28.2 ± 8.4	**<0.001**
Energy intake (kcal/d)	1950.1 ± 248.5	1943.9 ± 242.2	1956.3 ± 255.0	0.618
Fiber (g/d)	17.4 ± 7.3	18.0 ± 7.1	16.8 ± 7.5	0.093
Carbohydrates (g/d)	275.2 ± 20.2	274.8 ± 19.5	275.5 ± 20.8	0.731
Protein (g/d)	61.8 ± 13.8	61.5 ± 13.6	62.1 ± 14.1	0.651
Fat (g/d)	66.9 ± 14.0	66.5 ± 13.7	67.3 ± 14.3	0.572
Categorical variables				
SES				0.559
1	82 (20.5%)	40 (20.0%)	42 (21.0%)	
2	77 (19.3%)	36 (18.0%)	41 (20.5%)	
3	68 (17.0%)	34 (17.0%)	34 (17.0%)	
4	98 (24.5%)	46 (23.0%)	52 (26.0%)	
5	75 (18.8%)	44 (22.0%)	31 (15.5%)	
Income level				0.070
1	102 (25.5%)	62 (31.0%)	40 (20.0%)	
2	109 (27.3%)	47 (23.5%)	62 (31.0%)	
3	87 (21.8%)	42 (21.0%)	45 (22.5%)	
4	102 (25.5%)	49 (24.5%)	53 (26.5%)	
Smoking (yes)	83 (20.8%)	49 (24.5%)	34 (17.0%)	0.064
Supplement use (yes)	144 (36.0%)	83 (41.5%)	61 (30.5%)	**0.022**
Infertility etiology (among infertile only)				
PCOS-related	–	–	86 (43.0%)	–
Ovulatory dysfunction (non-PCOS)	–	–	52 (26.0%)	–
Unexplained	–	–	41 (20.5%)	–
Other causes (stage I–II endometriosis, cervical factor, combined mild factors)	–	–	21 (10.5%)	–
Ovarian reserve				
AMH (ng/mL)	2.62 ± 1.45	3.03 ± 1.52	2.21 ± 1.27	**<0.001**
AFC (*n*)	12.33 ± 4.95	14.15 ± 4.96	10.52 ± 4.22	**<0.001**

Data presented as mean ± SD or *n* (%). *p*-values from independent *t*-test or Chi-squared test. Bold values denote statistical significance (*p* < 0.05).

**Table 2:** Linear regression: DPI and biomarkers of oxidative stress/inflammation.

Biomarker	Crude model	*p*	Adjusted model	*p*
	β (95% CI)		β (95% CI)	
MDA	−0.042 (−0.061, −0.023)	**<0.001**	−0.041 (−0.061, −0.022)	**<0.001**
HOMA-IR	−0.044 (−0.063, −0.025)	**<0.001**	−0.044 (−0.063, −0.024)	**<0.001**
hs-CRP	−0.025 (−0.046, −0.004)	**0.019**	−0.025 (−0.046, −0.004)	**0.021**
Interleukin-6	−0.013 (−0.025, −0.0003)	**0.045**	−0.014 (−0.027, −0.001)	**0.030**
TNF-*α*	−0.004 (−0.024, 0.016)	0.690	−0.006 (−0.026, 0.015)	0.575
TAC	0.005 (−0.006, 0.015)	0.361	0.005 (−0.005, 0.016)	0.313
SOD	0.004 (−0.001, 0.010)	0.145	0.004 (−0.001, 0.010)	0.142
GPx	0.055 (−0.059, 0.168)	0.344	0.073 (−0.041, 0.188)	0.209
Catalase	−0.023 (−0.194, 0.149)	0.795	0.001 (−0.173, 0.175)	0.993

Adjusted for age, BMI, energy intake, physical activity, smoking, supplement use, SES. Bold values denote statistical significance (*p* < 0.05).

## Results

### Study participants

A total of 400 women (200 fertile, 200 infertile) were included in the final analysis. The mean age of participants was 31.7 ± 5.0 years, with no significant difference between fertile and infertile groups (31.4 ± 4.6 vs. 32.0 ± 5.3 years; *p* = 0.235). Among the 200 infertile women, the distribution of infertility etiologies was as follows: PCOS related infertility (*n* = 86, 43.0%), ovulatory dysfunction not meeting PCOS criteria (*n* = 52, 26.0%), unexplained infertility (*n* = 41, 20.5%), and other causes (*n* = 21, 10.5%; including stage I–II endometriosis [*n* = 12], cervical factor [*n* = 6], and combined mild factors [*n* = 3]). The mean DPI did not differ significantly across etiological subgroups (PCOS: 27.9 ± 8.1; ovulatory dysfunction: 28.5 ± 8.6; unexplained: 28.3 ± 8.4; other: 28.0 ± 8.9; *p* = 0.93), suggesting that the inverse association between DPI and infertility was not driven by a single diagnostic category. Compared to fertile women, infertile women had significantly higher BMI (27.5 ± 4.1 vs. 26.4 ± 3.8 kg/m^2^; *p* = 0.006), larger waist circumference (90.6 ± 10.1 vs. 87.3 ± 9.2 cm; *p* < 0.001), and lower Dietary Phytochemical Index (28.2 ± 8.4 vs. 34.1 ± 9.8; *p* < 0.001). Infertile women were also significantly less likely to use dietary supplements (30.5% vs. 41.5%; *p* = 0.022). Fertile women showed a non-significantly higher smoking prevalence (24.5% vs. 17.0%, *p* = 0.064). While adjusted models controlled for smoking, residual confounding from unmeasured lifestyle factors correlated with both smoking and diet cannot be excluded. No significant differences were observed between groups in age, physical activity, energy intake, dietary fiber, carbohydrate, protein, or fat intake, socioeconomic status, income level, or smoking status (all *p* > 0.05) (Table 1).

### Characteristics across DPI tertiles

When participants were stratified by infertility status and DPI tertiles, most baseline characteristics were well balanced across tertiles within both the fertile and infertile groups, supporting the comparability of these groups (Table 3). No other significant differences across tertiles were observed for demographic, anthropometric, dietary, or lifestyle variables in either group (all *p* > 0.05), indicating that potential confounding factors were evenly distributed across DPI categories.

**Table 3:** Characteristics across DPI tertiles by infertility status.

Characteristic	Fertile (*n* = 200)			*p*	Infertile (*n* = 200)			*p*
	T1 (*n* = 49)	T2 (*n* = 55)	T3 (*n* = 96)		T1 (*n* = 85)	T2 (*n* = 78)	T3 (*n* = 37)	
Continuous								
Age (y)	31.2 ± 4.9	31.9 ± 4.1	31.3 ± 4.8	0.713	32.3 ± 5.9	32.1 ± 4.8	31.2 ± 4.7	0.565
BMI (kg/m^2^)	26.1 ± 3.7	26.1 ± 4.2	26.7 ± 3.6	0.489	27.5 ± 4.0	27.4 ± 4.2	27.8 ± 4.3	0.913
Waist (cm)	86.8 ± 10.2	86.5 ± 8.9	88.0 ± 8.9	0.575	95.94 ± 8.7	87.47 ± 9.7	84.59 ± 8.04	**<0.001**
PA (MET)	1,504 ± 523	1,546 ± 526	1,440 ± 459	0.431	1,511 ± 433	1,560 ± 509	1,459 ± 545	0.563
Energy (kcal)	1943 ± 293	1927 ± 254	1954 ± 207	0.816	1937 ± 225	1957 ± 262	2000 ± 302	0.454
Fiber (g/d)	18.1 ± 7.8	18.2 ± 7.5	17.8 ± 6.7	0.950	17.3 ± 6.6	16.7 ± 7.8	15.7 ± 8.7	0.540
Carbs (g/d)	275.9 ± 24.8	273.7 ± 19.0	274.9 ± 16.8	0.857	274.0 ± 19.8	275.3 ± 20.6	279.4 ± 23.6	0.419
Protein (g/d)	61.8 ± 16.2	60.5 ± 13.8	61.9 ± 12.1	0.810	60.5 ± 12.6	62.5 ± 14.1	65.0 ± 16.8	0.267
Fat (g/d)	65.8 ± 15.8	65.6 ± 15.0	67.4 ± 11.7	0.691	66.5 ± 12.3	67.3 ± 15.3	69.2 ± 16.7	0.644
Food groups (servings/d)								
Fruits	1.21 ± 0.57	1.90 ± 0.70	2.60 ± 0.80	<0.001	1.06 ± 0.49	1.45 ± 0.63	2.17 ± 0.69	<0.001
Vegetables	1.70 ± 0.80	2.40 ± 1.06	3.35 ± 1.04	<0.001	1.75 ± 0.69	2.19 ± 0.78	2.93 ± 0.91	<0.001
Legumes	0.32 ± 0.20	0.54 ± 0.33	0.81 ± 0.42	<0.001	0.32 ± 0.17	0.42 ± 0.31	0.69 ± 0.46	<0.001
Nuts/seeds	0.22 ± 0.20	0.37 ± 0.25	0.78 ± 0.42	<0.001	0.23 ± 0.18	0.29 ± 0.23	0.67 ± 0.40	<0.001
Whole grains	0.80 ± 0.47	1.17 ± 0.55	1.94 ± 0.75	<0.001	0.69 ± 0.43	1.07 ± 0.53	1.44 ± 0.73	<0.001
Categorical (%)								
Smoking (yes)	26.5	21.8	25.0	0.845	18.8	15.4	16.2	0.835
Supplement (yes)	32.7	43.6	44.8	0.348	31.8	29.5	29.7	0.945
SES (4–5)	55.1	49.1	37.5	0.116	44.7	35.9	45.9	0.451
Income (3–4)	44.9	49.1	43.8	0.833	41.2	56.4	51.4	0.152

Data are mean ± SD or %. *p*-values from one-way ANOVA or Chi-squared test within each group. Bold values denote statistical significance (*p* < 0.05).

As shown in Table 3, higher DPI tertiles were associated with progressively greater consumption of fruits, vegetables, legumes, nuts/seeds, and whole grains in both fertile and infertile women (all *p* for trend <0.001). For example, fertile women in the highest DPI tertile consumed 2.60 ± 0.80 servings of fruits and 3.35 ± 1.04 servings of vegetables daily, compared with 1.21 ± 0.57 and 1.70 ± 0.80 servings, respectively, in the lowest tertile. Similar dose–response patterns were observed among infertile women. These data confirm that DPI reflects meaningful differences in intake of phytochemical-rich plant foods.

### DPI and biomarkers of oxidative stress and inflammation

In linear regression analyses (Table 2), DPI was significantly inversely associated with several biomarkers of oxidative stress and inflammation in both crude and fully adjusted models. To aid clinical interpretation, the beta coefficients represent the change in the natural log transformed biomarker per 1 unit increase in DPI. For example, the beta of −0.041 for MDA (95% CI: −0.061, −0.022) corresponds to an approximate 4.0% lower MDA level per 10 unit increase in DPI. Prior to multivariable adjustment, infertile women had significantly higher levels of MDA (5.2 ± 1.4 vs. 4.1 ± 1.1 μmol/L, *p* < 0.001), HOMAIR (3.8 ± 1.6 vs. 2.5 ± 1.1, *p* < 0.001), hsCRP (3.2 ± 1.9 vs. 1.8 ± 1.2 mg/L, *p* < 0.001), and IL6 (2.9 ± 1.3 vs. 2.1 ± 0.9 pg./mL, *p* < 0.001) compared with fertile women, consistent with the hypothesized direction of association. In the fully adjusted models (adjusted for age, BMI, energy intake, physical activity, smoking, supplement use, and socioeconomic status), each unit increase in DPI was associated with significantly lower levels of MDA (*β* = −0.041; 95% CI: −0.061, −0.022; *p* < 0.001), HOMA-IR (*β* = −0.044; 95% CI: −0.063, −0.024; *p* < 0.001), hs-CRP (*β* = −0.025; 95% CI: −0.046, −0.004; *p* = 0.021), and interleukin-6 (*β* = −0.014; 95% CI: −0.027, −0.001; *p* = 0.030). No significant associations were found between DPI and TNF-*α* (*p* = 0.575), TAC (*p* = 0.313), SOD (*p* = 0.142), GPx (*p* = 0.209), or catalase (*p* = 0.993) in adjusted models.

### DPI and infertility risk

Logistic regression analysis revealed a strong, independent inverse association between DPI and infertility (Table 4). In the crude model, each 1 unit increase in DPI was associated with 7% lower odds of infertility (OR = 0.931; 95% CI: 0.909–0.953; *p* < 0.001). To facilitate clinical interpretation, a 10 unit increase in DPI—approximately the difference between the lowest and highest tertiles—was associated with a 48% lower odds (OR = 0.931^10 ≈ 0.48). This association remained virtually unchanged after adjustment for age and BMI (OR = 0.929; 95% CI: 0.907–0.952; *p* < 0.001) and in the fully adjusted model including all covariates (OR = 0.926; 95% CI: 0.903–0.950; *p* < 0.001).

**Table 4:** Logistic regression: DPI and infertility risk.

Model	Exposure	OR (95% CI)	*p*-value
Model 1: Crude	DPI (continuous)	0.931 (0.909–0.953)	**<0.001**
Model 2: + Age, BMI	DPI (continuous)	0.929 (0.907–0.952)	**<0.001**
Model 3: Age + BMI + total energy intake	DPI (continuous)	0.926 (0.903–0.950)	**<0.001**
Model 4: Tertiles (crude)	T1 (Low)	1.00 (ref)	–
	T2 (Medium)	0.818 (0.500–1.338)	0.423
	T3 (High)	0.222 (0.132–0.373)	**<0.001**
Model 5: Tertiles (fully adjusted)	T1 (Low)	1.00 (ref)	–
	T2 (Medium)	0.784 (0.469–1.312)	0.354
	T3 (High)	0.205 (0.119–0.352)	**<0.001**
Trend test (fully adjusted)	DPI per tertile median	0.920 (0.893–0.946)	**<0.001**

Model 1: Crude. Model 2: Age + BMI. Model 3: Age + BMI + total energy intake. Model 5 (Fully adjusted): Model 3 + physical activity + smoking + supplement use + SES. Bold values denote statistical significance (*p* < 0.05).

When DPI was analyzed as tertiles, a clear dose–response relationship was observed. In the fully adjusted model, women in the highest DPI tertile had an 80% lower odds of infertility compared to those in the lowest tertile (OR = 0.205; 95% CI: 0.119–0.352; *p* < 0.001). The middle tertile showed a non-significant associated with lower odds (OR = 0.784; 95% CI: 0.469–1.312; *p* = 0.354). The test for linear trend across tertile medians was statistically significant (OR = 0.920 per tertile increase; 95% CI: 0.893–0.946; *p* < 0.001), confirming a significant dose–response gradient. The fully adjusted logistic regression model demonstrated good calibration (Hosmer–Lemeshow goodness of fit test: χ^2^ = 6.82, df = 8, *p* = 0.556) and moderate discrimination (area under the receiver operating characteristic curve [AUC] = 0.78, 95% CI: 0.73–0.83). The AUC remained stable after bootstrap validation with 200 resamples (optimism corrected AUC = 0.76), indicating acceptable model performance for a nutritional epidemiology study.

Subgroup analyses showed consistent inverse associations between DPI (per 10-unit increase) and infertility across BMI categories (normal: OR 0.44, overweight: 0.47, obese: 0.51), smoking status (never: 0.46, current: 0.49), and insulin resistance (HOMA-IR < 2.5: 0.45, ≥2.5: 0.48). Interaction tests were non-significant (all *p* > 0.68), indicating no effect modification by these factors (Supplementary Table S1).

### Predicted probability of infertility

Figure 2 illustrates the adjusted predicted probability of infertility across the range of DPI values. The probability of infertility decreased progressively from approximately 65% at DPI values of 10 to approximately 25% at DPI values of 50, demonstrating a continuous inverse relationship between DPI and infertility risk.

### Mediation analysis

In exploratory mediation analyses (acknowledging cross-sectional limitations), DPI was significantly associated with lower HOMA-IR, MDA, hs-CRP, and IL-6 (path a), but none of these biomarkers were significantly associated with infertility (path b). All indirect effects were non-significant (proportion mediated: 0.01–12.6%; all 95% CIs included zero), while the direct effect of DPI remained significant (OR ≈ 0.93 per 1-unit DPI; *p* < 0.001). These exploratory findings do not rule out biologically meaningful mediation, as single-time-point circulating biomarkers may not reflect local oxidative stress, measurement error can attenuate effects, and the case–control design precludes causal inference. Results are hypothesis-generating; prospective studies with repeated biomarker measurements are needed (Table 5).

**Table 5:** Mediation analysis of the association between DPI and infertility.

Mediator	Path a^1^ β (SE)	Path b^2^ OR (95% CI)	Indirect effect^3^ (95% CI)	Direct effect^2^ OR (95% CI)	% Mediated^4^
HOMA-IR	0.04 (0.01)	1.02 (0.90–1.16)	0.00 (−0.01, 0.01)	0.93 (0.90–0.95)	12.6%
MDA	0.04 (0.01)	1.00 (0.89–1.12)	0.00 (−0.01, 0.01)	0.93 (0.90–0.95)	4%
hs-CRP	0.03 (0.01)	1.00 (0.89–1.12)	0.00 (−0.00, 0.01)	0.93 (0.90–0.95)	1.8%
Interleukin-6	0.01 (0.01)	1.09 (0.95–1.24)	0.00 (−0.01, 0.00)	0.92 (0.90–0.95)	1.98%
TNF-α	0.01 (0.01)	0.99 (0.88–1.11)	0.00 (−0.00, 0.00)	0.93 (0.90–0.95)	0.07%
TAC	0.01 (0.01)	0.97 (0.85–1.09)	0.00 (−0.00, 0.00)	0.93 (0.90–0.95)	0.01%

*p* < 0.05, *p* < 0.001. ^1^Path a: DPI → Mediator (adjusted linear regression). ^2^Path b and Direct effect: Adjusted logistic regression. ^3^Indirect effect (a × b) from 1,000 bootstrap replications. ^4^% Mediated = |indirect|/|total (−0.0769) | × 100.

In summary, higher DPI was strongly and independently associated with lower odds of female infertility in a dose–response manner. Women in the highest DPI tertile had 80% lower odds compared to those in the lowest tertile. For context, this corresponds to a reduction in predicted probability from approximately 55% in the lowest tertile to approximately 18% in the highest tertile, based on the fully adjusted model (Figure 2). However, odds ratios overestimate risk differences when the outcome is common; readers should focus on the OR as a measure of association, not absolute risk. DPI was also significantly inversely associated with MDA, HOMA-IR, hs-CRP, and IL-6. However, bootstrap mediation analyses revealed that none of these biomarkers showed statistically significant indirect effects. However, due to the cross-sectional design, these findings do not rule out biological mediation; they simply indicate that the selected circulating biomarkers measured at a single time point did not statistically account for the association. These findings indicate that a phytochemical-rich diet may are associated with lower odds of infertility through mechanisms largely independent of the circulating oxidative stress and inflammatory biomarkers assessed in this study.

## Discussion

This case–control study demonstrated a robust, independent, and dose-dependent inverse association between the DPI and female infertility. Women in the highest tertile of DPI had 80% lower odds of infertility relative to those in the lowest tertile, after comprehensive adjustment for potential confounders. Each 10-unit increase in DPI was associated with a 48% lower odds of infertility, and a significant linear trend across tertiles confirmed a graded protective relationship. These findings identify DPI as a novel nutritional marker strongly linked to reproductive health. Our results are consistent with prior work showing that higher intake of fruits, vegetables, and whole grains is associated with improved fecundability and IVF outcomes, to our knowledge, this is the first study to report a dose–response relationship between a composite dietary phytochemical density index and clinically diagnosed female infertility.

Our findings build on existing research linking dietary patterns to fertility. Although adherence to a Mediterranean diet and high intake of fruits, vegetables, and whole grains have been associated with better fecundability and ART outcomes, this study is the first to use the DPI to quantify dietary phytochemical density in relation to clinically diagnosed infertility. Unlike other indices—the Dietary Inflammatory Index (DII) reflects inflammation but not phytochemicals, the Healthy Eating Index (HEI) follows general guidelines without emphasizing phytochemical-dense foods (e.g., potatoes offer limited phytochemical diversity), and plant-based indices lack weighting by phytochemical density—the DPI directly captures phytochemical intake, which may confer antioxidant and endocrine benefits beyond inflammation reduction (11, 12, 20, 24). The observed association for DPI (OR 0.48 per 10-unit increase, equivalent to 52% lower odds) is substantial and exceeds the 30–40% reduction in infertility odds reported for high Mediterranean diet adherence in a recent meta-analysis (12). This suggests that the DPI may identify additional protective components of plant-based diets, potentially reflecting synergistic effects of multiple phytochemical compounds on the reproductive system (7, 13, 24).

Although DPI was inversely associated with MDA, HOMA-IR, hs-CRP, and IL-6—consistent with known anti-oxidative and anti-inflammatory properties of phytochemicals (7, 13, 25)—these biomarkers did not statistically mediate the DPI–infertility association. The lack of association with enzymatic antioxidants (SOD, GPx, catalase) despite an inverse association with MDA suggests that DPI may influence non-enzymatic antioxidant capacity rather than upregulating endogenous enzyme activity (7, 18). Previous studies in non-fertile populations reported similar inverse associations, supporting the internal validity of our findings (4, 6, 8). However, we caution against concluding that oxidative stress and inflammation are mechanistically unimportant. Circulating biomarkers measured at a single time point may not reflect chronic exposure, local ovarian/endometrial oxidative stress, or specific modifications (e.g., oocyte DNA damage) most relevant to fertility. Measurement error (especially for hs-CRP and IL-6) can also attenuate indirect effects. The inverse associations in Table 2 confirm engagement of these pathways, but they may not be the primary mediators. Definitive conclusions require prospective studies with repeated and local (follicular fluid) biomarker measurements (26). Unlike earlier cross-sectional studies that lacked formal mediation tests, our rigorous bootstrap approach provides stronger evidence against a simple mediation pathway.

Several explanations may account for the absence of mediation. First, dietary phytochemicals might act locally within ovarian follicles, endometrium, or the hypothalamic–pituitary-ovarian (HPO) axis, effects not captured by circulating MDA, hsCRP, or HOMAIR. Beyond their antioxidant actions, phytochemicals—particularly polyphenols and flavonoids—have been shown to accumulate in ovarian tissue and follicular fluid, where they exert multiple effects relevant to fertility: (a) promoting folliculogenesis by upregulating the PI3K/Akt signaling pathway and reducing granulosa cell apoptosis; (b) enhancing oocyte meiotic competence and mitochondrial function, preserving mitochondrial membrane potential and ATP production necessary for fertilization and early embryonic development; (c) modulating steroid hormone synthesis by affecting aromatase activity and gonadotropin receptor expression; (d) improving endometrial vascular function and receptivity through endothelial nitric oxide synthase activation and regulation of adhesion molecules; and (e) inducing epigenetic modifications, including DNA methylation changes and histone acetylation, that may influence gene expression related to reproduction. These local effects would not be reflected in our circulating systemic biomarkers, potentially explaining the absence of statistical mediation (13, 20, 25). Second, we assessed mediators at a single time point, potentially missing dynamic changes in oxidative stress and inflammation across the menstrual cycle or after meals. Third, phytochemicals may act via unmeasured pathways—gut microbiota modulation, epigenetic changes, or hormone receptor binding. Indeed, gut microbes convert phytochemicals into bioactive metabolites that could influence distant reproductive organs. Finally, the cross-sectional mediation analysis limits causal inference about temporal pathways (5, 6, 12).

Strengths include the well-characterized sample, a validated dietary index quantifying phytochemical density, and rigorous adjustment for confounders (energy intake, physical activity, socioeconomic status). Bootstrap mediation with 1,000 replications provided robust indirect effect estimates. The dose–response gradient reinforces biological plausibility.

Several limitations warrant mention. The case–control design precludes causal inference and is subject to recall bias. Diet was assessed once via FFQ, missing long-term habits, seasonal variation, and phytochemical bioavailability (which depends on food preparation and individual metabolism). The modest sample size limited power for subgroup and mediation analyses. Generalizability is constrained by the single geographic and cultural context; DPI sources vary across populations. Unmeasured confounding remains possible. We did not collect data on partner fertility status, history of sexually transmitted infections, prior miscarriages, or detailed socioeconomic measures (beyond SES and income level). These factors could influence both dietary patterns and infertility risk, and their absence may introduce residual confounding. Residual confounding from unmeasured lifestyle factors (e.g., stress, environmental exposures, other health-promoting behaviors) may persist despite adjustment, and women with higher DPI might engage in such behaviors not fully captured. Additionally, our DPI calculation excluded non-caloric phytochemical sources (tea, coffee, herbs, spices), potentially underestimating total exposure. Reverse causation is possible, as infertility could influence dietary recall, though we assessed diet over the 12 months preceding enrollment and recruited cases before treatment initiation. Mediation analyses in case–control designs may be biased by unmeasured confounders and assume a rare outcome; thus, indirect effects are exploratory. The DPI captures overall phytochemical density but does not differentiate among classes with distinct bioactivities. Finally, we excluded women with tubal factor infertility and severe endometriosis to reduce etiological heterogeneity, which strengthens internal validity for detecting diet-metabolism associations but limits generalizability to these important subtypes. Future studies specifically examining these subgroups are warranted.

Clinically, advising a phytochemical-rich diet (fruits, vegetables, legumes, whole grains, nuts, herbs) is reasonable as a low-risk, healthful pattern. Our observational findings show a strong association with lower infertility odds, but causality cannot be inferred. A practical 10-unit DPI increase can be achieved by replacing ≈200 kcal of refined grains with berries, cruciferous vegetables, and legumes or nuts. The DPI offers a quantitative research framework, but direct clinical efficacy is not established. Future prospective cohorts and randomized trials are needed. Mechanistic studies should include: (1) longitudinal biomarker profiling across the menstrual cycle; (2) targeted metabolomics of phytochemical metabolites; (3) assessment of follicular fluid biomarkers; and (4) integration of gut microbiome analyses with reproductive outcomes.

## Conclusion

In conclusion, this observational case–control study provides novel evidence that a higher DPI is strongly and independently associated with lower odds of clinically diagnosed infertility—extending prior work that focused on fecundability or ART outcomes—with a clear dose–response pattern. Notably, it also integrates a mediation analysis of multiple circulating biomarkers within a single cohort. However, due to the inherent limitations of the case–control design (residual confounding, recall bias, inability to establish temporality), causality cannot be inferred; the findings are hypothesis-generating. Dietary phytochemical density, rather than isolated nutrients, may represent a key fertility parameter. Practically, preconception counseling should emphasize whole plant foods over supplements. Future prospective cohort validation, randomized trials of phytochemical-rich dietary interventions, and mechanistic studies at the ovarian and endometrial level are needed to confirm and extend these findings.

## Data Availability

The original contributions presented in the study are included in the article/supplementary material, further inquiries can be directed to the corresponding author.
