# Supplementary material for: Higher dietary phytochemical index is associated with lower odds of infertility: a case–control study
Source: Front Nutr. 2026 Jul 10;13:1865952. doi: 10.3389/fnut.2026.1865952 (PMC13395712; doi:10.3389/fnut.2026.1865952)
Supplement: Supplementary file 1 [file Table_1.docx]

| supplementary Table 1. Subgroup analysis: Association between DPI (per 10‑unit increase) and infertility | | | |
| --- | --- | --- | --- |
| Subgroup | n (%) | OR (95% CI) per 10‑unit DPI | P for interaction |
| BMI category |  |  | 0.68 |
| Normal (18.5–24.9) | 142 (35.5%) | 0.44 (0.32–0.60) |  |
| Overweight (25.0–29.9) | 168 (42.0%) | 0.47 (0.35–0.63) |  |
| Obese (≥30) | 90 (22.5%) | 0.51 (0.37–0.70) |  |
| Smoking status |  |  | 0.74 |
| Never smoker | 317 (79.3%) | 0.46 (0.36–0.59) |  |
| Current smoker | 83 (20.8%) | 0.49 (0.33–0.72) |  |
| Insulin resistance (HOMA‑IR ≥2.5) |  |  | 0.81 |
| No | 211 (52.8%) | 0.45 (0.34–0.60) |  |
| Yes | 189 (47.2%) | 0.48 (0.36–0.64) |  |

*ORs adjusted for age, BMI (except for BMI‑stratified models), total energy intake, physical activity, smoking (except for smoking‑stratified), supplement use, and SES. Interaction tests from fully adjusted models including DPI × subgroup term.*
